# Predictors of pre- and post-competition affective states in male martial artists: a multilevel interactional approach

**DOI:** 10.1111/j.1600-0838.2009.01006.x

**Published:** 2011-02

**Authors:** E Cerin, A Barnett

**Affiliations:** 1 USDA/ARS Children's Nutrition Research Center, Baylor College of MedicineHouston, Texas, USA; 2 School of Health and Human Performance, Central Queensland UniversityRockhampton, Qld, Australia; 3 Centre of Excellence for Applied Sport Science Research, Queensland Academy of SportNathan, Qld, Australia

**Keywords:** experience sampling method, prospective study, personality, emotions, competitive trait anxiety

## Abstract

The aims of this study were to examine (a) the effects of competition-related and competition-extraneous concerns on affective states; (b) the relationships of primary and secondary appraisal with affective states and (c) the main and moderating effects of personality traits on pre- and post-competition affects. Thirty-nine male elite martial artists were assessed on 12 affective states, concerns and dimensions of primary and secondary appraisal at five random times a day across 1 week before and 3 days after a competition. On the competition day, they were assessed 1 h before and immediately after the contest. Competitive trait anxiety, neuroticism and extraversion were measured at the start of the study. The competition was the most significant and stressful event experienced in the examined period and had a pervasive influence on athletes' affective states. All examined appraisal and personality factors were somewhat associated with pre- and post-competition affective states. Competitive trait anxiety was a key moderator of the relationship between cognitive appraisal and affective states. This study supports the idea that cognitive appraisal and situational and personality factors exert main and interactive effects on athletes' pre- and post-competition affects. These factors need to be accounted for in planning of emotion regulation interventions.

It is generally maintained that affective states or affects, a generic concept subsuming emotions, moods and feelings, can impact on athletic performance (Hanin, 2000). Affects also provide important information on the athlete–competition relationship in terms of personal importance attributed to a competitive event and perceived ability to cope with it (Lazarus, 1999; Uphill & Jones, 2007;). An examination of personal, situational and cognitive factors associated with athletes' pre- and post-competition affects can aid the planning and implementation of performance and wellbeing enhancement programs.

## Defining affective states

This paper focuses on pre- and post-competition emotions and moods. These two concepts differ along several dimensions: duration, reference to an object, origin and intensity (Vallerand & Blanchard, 2000). Emotions are defined as sudden, short-lasting reactions to a specific, identifiable actual or imagined event leading to physiological and experiential changes and object-focused behavior. Many emotion theorists maintain that there are a number of primary discrete emotions (also called “basic” or “fundamental”) that underlie people's emotional lives (Izard, 1991; Ekman, 1994; Lazarus, 1999;). Basic emotions are typified by distinctive antecedent events, physiology, emotional expressions, appraisal patterns (one's evaluation of the importance and desirability of an event and the ability to cope with it), relational action tendencies (e.g., approach or avoidance) and presence in all cultures and primates (Ekman, 1994). The study of basic emotions in a sporting context is important because they are affective phenomena comparable across individuals, cultures and settings. Also, they have been shown to be useful predictors of self-referenced performance and reliable indicators of athletes' appraisals of a sport-related event (Cerin, 2003).

In this study, basic emotions were conceptualized according to Izard's (1991) differential emotions theory (DET), which encompasses 11 basic emotions: anger, contempt, disgust, enjoyment, fear, guilt, interest–excitement, sadness, shame, shyness and surprise (for details see Cerin & Barnett, 2006). We adopted this specific model of basic emotions because it had been successfully used in a previous study on individual-sport athletes (Cerin, 2003) and it was one of the few available models to include shame and shyness, two salient pre- (Cerin, 2003) and post-competition emotions (Wilson & Kerr, 1999).

Compared with emotions, moods are usually longer lasting. They are more diffuse, have no apparent triggering stimulus, and are not associated with object-focused behavior and emotional expressions (Vallerand & Blanchard, 2000). A distinction between moods and emotions can clarify athletes' psychological response to a competitive event, i.e., the extent to which affective reactions to a competition are ‘conscious’ event-related (emotions), or more subtle and individual-related, experiences (moods). Such knowledge is important for the planning of psychological interventions aimed at optimizing athletes' pre- and post-competition psychological states, as mood and emotion regulation strategies differ. For example, compared with emotion regulation, mood regulation is more concerned with altering affective experience than affective behavior (Gross, 2006). Also, mood and emotions may influence athletic performance in distinct ways (Mellalieu, 2003) so it is important to examine their unique contribution.

## A model of competition-related affective states

To explain athletes' affective reactions to competitive events, a process-based interactional model of competitive stress has been proposed (Cerin et al., 2000). This model integrates Lazarus' interactional model of stress (Lazarus, 1999) with research and theories on competition-related affects (Hanin, 2000). It is postulated that competition demands, opportunities and constraints influence athletes' quality and intensity of affective response through the process of appraisal. In turn, affective states are proposed to influence athletes' behavior and performance. The model acknowledges that affective reactions to competition vary considerably across individuals and competitive events. These differential reactions are thought to be determined by personal (e.g., personality traits) and situational factors (e.g., environmental conditions and life events unrelated to the competition), and their interaction (Cerin et al., 2000). It is also postulated that affects, appraisal, coping strategies and some personal and situational variables change. Hence, competition and athletes' differential reaction to it are to be studied as a process that unfolds over time.

## Appraisal and affective states

Appraisal is defined as the perceived personal relevance (primary appraisal) of an event, and the ability to cope with it (secondary appraisal) (Lazarus, 1999). According to Smith and Lazarus (1993), there are three primary and three secondary appraisal dimensions. These are the primary appraisal dimensions of goal relevance, goal congruence and type of ego-involvement and the secondary appraisals of blame/credit, coping potential and future expectations (Lazarus, 1999). This study focused on all appraisal dimensions except for type of ego involvement (the type of goal at stake associated with an emotion-triggering event), which was not examined due to the intensive nature of the study (see “Study limitations” for details).

Goal relevance refers to the importance of the situation and is hypothesized to determine emotion intensity. Goal congruence refers to the perceived benefit, harm or threat related to a particular situation. This dimension determines the hedonic tone of one's emotions. Coping potential relates to whether an individual can control and improve the person–environment relationship. Perceived personal control over a source of concern (i.e., emotion-triggering event) is deemed to be positively associated with enjoyment (Weiner, 1985), interest, guilt (Weiner, 1985; Lazarus, 1999;) and self-hostility (Izard, 1991), but negatively associated with surprise (Weiner, 1985), anger, sadness, disgust (Weiner, 1985; Lazarus, 1999;), fear/anxiety (Lazarus, 1999) and shame (Thompson et al., 2004). Blame and credit depend on whether an attribution of accountability for a negative or positive event can be made and on how much control those accountable have over their actions. The locus of control (internal or external) influences the emotion that will be experienced. Thus, potentially controllable negative events attributed to the self can trigger guilt and self-hostility, while those attributed to others may evoke anger or disgust (Lazarus, 1999). Future expectations refer to the possibility that, in the future, things will improve or worsen for any reason (Lazarus, 1999). Negative expectations are likely to trigger negative emotions (e.g., sadness, shame and fear/anxiety), while positive expectations are likely associated with elevated interest/excitement and enjoyment (Izard, 1991; Lazarus, 1999;).

Several cross-sectional studies examined the relationships of dimensions of cognitive appraisals with pre- and post-competition affects (e.g., Hanton & Jones, 1997; Graham et al., 2002;). The problem with these cross-sectional studies is that they tend to confound trait and state factors affecting psychological phenomena and may give a significantly distorted picture of how appraisal and affects co-vary across time (Watson, 1988). A number of recent studies examined processual aspects of competitive stress (e.g., Owen et al., 2007; Uphill & Jones, 2007; Mellalieu et al., 2008; Nicholls et al., 2009;). However, no studies examined the magnitude of relationships between appraisal and affects using a mixed nomothetic–idiographic approach whereby the components of the stress process are frequently assessed on the same individuals over a period of time. The main strength of this type of approach is that it minimizes memory bias and allows a simultaneous analysis of inter-individual differences and intra-individual changes across time and the identification of intra- and inter-individual moderating factors.

## Interactive effects of personality traits and situational factors

The interactional model of competitive stress postulates that cognitive appraisal of, and the affective reaction to, a competitive event are influenced by personal (e.g., personality traits) and situational factors (e.g., proximity of a competition) and their interaction (Cerin et al., 2000). Research in mainstream psychology has found consistent relationships between negative affects and neuroticism and positive affects and extraversion (Costa & McCrae, 1992). In sport, competitive trait anxiety and neuroticism were found to be related to magnitude and/or temporal patterns of pre-competition and post-competition anxiety (Huband & McKelvie, 1986) and other pre-competition negative affects (Hassmén et al., 1998). Cerin (2004) observed significant interactive effects of neuroticism with competition proximity and negative affect on anxiety direction (whether anxiety is perceived as detrimental or facilitative to performance). Specifically, athletes with above-average neuroticism showed a significant worsening in anxiety direction as the competition neared. No such effect was found in those with below-average neuroticism. Furthermore, higher negative affects were predictive of poorer cognitive anxiety direction in athletes with high neuroticism. The opposite was observed for those low in neuroticism. Overall, these findings indicate that the study of personality traits can offer a more comprehensive understanding of athletes' emotional response to the competitive process and, at the same time, potentially provide an explanation for the differential effects of pre-competition affects on performance.

## The present study: testing assumptions of the interactional model of competitive stress

Several longitudinal studies have attempted to shed light on the process of competitive stress by examining the temporal course of competition-related affects and/or aspects of cognitive appraisal (e.g., Robazza et al., 2000; Mellalieu et al., 2008; Nicholls et al., 2009;). However, to date, no studies have quantified the extent to which changes/fluctuations in goal relevance, congruence and controllability impact on pre- and post-competition affective states, nor have they analyzed how appraisal dimensions and emotions associated with a competition compare to those arising from competition-extraneous events. Such knowledge can help gain a better and holistic understanding of athletes' affective experience in temporal proximity of a competitive event. It is also unknown if competition-specific (e.g., competitive trait anxiety) and global personality traits, such as neuroticism and extraversion, moderate the temporal course of discrete affective states pre- and post-competition, and whether they can explain individual differences in the associations between appraisal dimensions and affects. Given that athletes' affective experiences in the days leading and following a competition are influenced by competition-related and competition-extraneous events, it is important to simultaneously examine the effects of both sport specific and generic personality traits on cognitive appraisals and associated affects.

To elucidate the above issues, an 11-day study adopting a mixed nomothetic–idiographic approach, using the Experience Sampling Methodology (ESM; Hormuth, 1986; Cerin et al., 2001;), which permits the monitoring of the spontaneous flow of daily affective and cognitive experiences in the athletes' habitual environment, was conducted (Cerin & Barnett, 2006). During the study, athletes were not explicitly asked competition-related questions in order to obtain more realistic and spontaneous information on the athlete–competition relationship and the role and subjective importance of the competition as compared with events in other life domains. By asking participants to report affective states and concerns associated with them (if any), we attempted to distinguish emotions (an affective state evoked by an identifiable cause) from moods (affective states with no apparent triggering stimulus). Presence vs absence of an identifiable triggering event was the sole criterion used to distinguish moods from emotions because its assessment is straightforward and the presence of an identifiable triggering stimulus is, by definition, a necessary condition for an emotion to occur (Vallerand & Blanchard, 2000). In contrast, the presence of emotional expressions and object-focused behaviors are not necessary conditions as they can be suppressed (Izard, 1991). Finally, there is substantial overlap between the intensity and duration of moods and emotions (Lane & Terry, 2000; Vallerand & Blanchard, 2000;), making it difficult to use them as criteria for the differentiation of these two affective phenomena.

While two companion papers explored temporal patterns of pre- and post-competition sources of concerns and affects (Cerin & Barnett, 2006) and affective spill-over across competition-related and competition-extraneous concerns (Cerin & Barnett, unpublished observation), respectively, this paper focused on the relationships between affects and their theoretical determinants. Based on the extant literature and interactional models of general (Lazarus, 1999) and competitive stress (Cerin et al., 2000), several hypotheses were formulated. These are presented below.

### Associations of appraisal dimensions with pre- and post-competition affects

Events are postulated to trigger specific emotions through appraisals (Smith & Lazarus, 1993; Lazarus, 1999;). Thus, we hypothesized that the pattern of associations of pre- and post-competition emotions with the appraisal dimensions would follow those predicted by Lazarus (1999) and Weiner (1985) (see introductory section “Appraisal and affective states”).

### Differences in emotions and cognitive appraisals between competition-related and competition-extraneous concerns

Given the athletes' likely high levels of importance attributed to the competition and high levels of perceived sport competence, it was hypothesized that competition-related concerns would, in general, be perceived as more important and controllable than their counterparts. Competition is usually associated with a mix of threat and challenge appraisals (Cerin, 2004). Thus, it was expected that, before the contest, competition-related concerns would elicit higher levels of fear, interest and enjoyment than competition-extraneous concerns. Post-competition, competition-related concerns were expected to elicit higher levels of outcome-dependent emotions (e.g., enjoyment, sadness and self-hostility) than competition-extraneous concerns (Weiner, 1985; Wilson & Kerr, 1999;). Pre- and post-competition affects have been shown to depend on the proximity of the competition (Robazza et al., 2000; Cerin et al., 2001; Gaudreau et al., 2002;) implying that the difference in level of positive and negative emotions elicited by competition-related vs competition-extraneous concerns would increase as the competition approached and gradually subside after the contest.

### Interaction effects of personality traits with appraisals and proximity of competition

Competitive trait anxiety is a situation-specific trait. Hence, it was expected to interact with the type of concern and predict higher levels of negative and lower levels of positive affects for competition-related as compared with competition-extraneous concerns. Because of higher levels of vulnerability to competition-related and/or general stressors, participants high on competitive anxiety and neuroticism were expected to show larger changes in levels of affects pre- and post-competition (Huband & McKelvie, 1986; Hassmén et al., 1998; Cerin, 2004;) and be more strongly influenced by performance appraisals at the competition than their counterparts (Rainey & Cunningham, 1988; Costa & McCrae, 1992;).

## Methods

### Participants

The study targeted individual, contact and subjectively scored sports (Tae Kwon Do and Karate) because there is evidence that these types of sport evoke greater changes in pre- and post-competition affects than other types of sport (Martens et al., 1990). Twenty-two male Tae Kwon Do and 22 male Karate practitioners participating in a major national competition were recruited for this study. Six participants competed at international and 38 at national level. Thirty-nine participants completed the 11 days of experience sampling. Five participants discontinued participation within 72 h of experience sampling either due to health problems or high-perceived participant's burden. No significant differences in personality and demographics were found between athletes who completed the study and those who did not.

The group of martial artists who completed the study ranged in age from 16 to 53 years (26.77±7.75) and had a mean training experience of 10.40 years (SD=6.47). Their mean perceived current performance was 3.72 (SD=0.65) on a five-point Likert scale ranging from 1 (*extremely poor*) to 5 (*excellent*). On average, they expected to perform slightly above their usual standard at the forthcoming competition. When compared with the norms for male American adults (Costa & McCrae, 1992), this group of athletes exhibited average neuroticism (52nd percentile) and above average extraversion (75th percentile). The sample had a mean level of competitive trait anxiety that corresponded to the 60th percentile of the norms for male wrestlers (Martens et al., 1990).

### Instruments

#### Demographic questionnaire (DQ)

Demographic information was obtained through a short questionnaire assessing age, training experience, level of participation and perceived current performance.

*The SCAT, Form A* (Martens et al., 1990) was used to measure competitive trait anxiety. The SCAT is a 15-item questionnaire gauging an individual's tendency to perceive competitive situations as threatening and to respond to these situations with elevated state anxiety. Participants are asked to indicate how they generally feel when they compete in sports and games and respond to each item using a three-point scale (*hardly ever, sometimes* and *often*). Scores on the SCAT range from 10 to 30. The SCAT is used extensively in sport psychology research, and has satisfactory test–retest reliability (*r*=0.61–0.95), and internal consistency (α=0.95–0.97) (Martens et al., 1990).

#### NEO PI-R, Form S: neuroticism and extraversion scale

The NEO PI-R is a self-report measure of the five major dimensions, or domains of personality (neuroticism, extraversion, openness, agreeableness and conscientiousness). Each personality factor is measured with a scale consisting of 48 items answered on a five-point scale from *strongly disagree* to *strongly agree*. For the purpose of this study, the participants were assessed on the personality domains of neuroticism and extraversion. This is because they are related to proneness to experience negative and positive affects and have been shown to moderate the temporal patterns of affective states in proximity of competition (Cerin, 2004). Specifically, neuroticism refers to the tendency to experience irrational ideas and negative emotions such as fear, shame, anger, guilt, sadness and disgust. It also entails poor ability to control impulses and cope with stress (Costa & McCrae, 1992). Extraversion is typified by sociability, preference for large groups and gatherings, assertiveness, optimism, excitement seeking and high activity levels. Internal consistency for the neuroticism and extraversion scales ranged from 0.89 to 0.92 (Costa & McCrae, 1992). Data on the validity of these factors are reported in the manual (Costa & McCrae, 1992).

*The DES-IV* (Izard, 1991) is a self-report instrument designed for the use and assessment of an individual's experience of fundamental emotions or patterns of emotions as conceptualized by the DET (Izard, 1991). The item content of the DES was derived from cross-cultural research on emotion expression labelling (Izard et al., 1993). The DES-IV comprises 12 three-item emotion subscales: interest-excitement, enjoyment, surprise, sadness, anger, disgust, contempt, fear, guilt, shame, shyness and self-hostility. The instructional set used in the study was “Read each statement and … indicate how you feel RIGHT NOW.” Several studies have contributed evidence for the construct validity of the DES scales (e.g., Izard et al., 1993; Youngstrom & Green, 2003;). The possible intensity scores on each subscale of the DES-IV range from 3 to 15. In this study, all subscales except for one (contempt with Cronbach's α 0.43) showed acceptable levels of reliability (Cronbach's α from 0.73 to 0.96). Hence, the contempt subscale was excluded from subsequent analyses. The DES-IV was previously found to be a good predictor of perceived functionality of affective states in relation to athletic performance and of recalled performance appraisals in individual sports (Cerin, 2003).

#### Assessment of sources of concern

Participants were asked to describe a positive or negative event, situation or thought (if any) that occurred in the interval before their last self-report and affected their current affective state. The reported sources of concern were coded according to the activity context with the categories competition-extraneous and competition-related. These categories were mutually exclusive. Interrater agreement between two independent coders was assessed for 761 events using Cohen's κ. Cohen' κ was 0.98 for competition-related sources of concern and 0.99 for competition-extraneous sources of concern. Participants also rated desirability, controllability and importance of the reported source of concern. Controllability and importance were rated on a seven-point Likert scale ranging from 1 (*not at all*) to 7 (*very much*). Desirability of the source of concern was defined as a dichotomous variable (e.g., desirable vs undesirable).

*Performance expectations and cognitive appraisal of performance at the competition* were measured on an 11-point Likert scale ranging from 0 (*very much below my usual standard*) to 10 (*very much above my usual standard*). Performance expectations were measured at the beginning of the study, whereas appraisal of performance at the competition was assessed immediately after the contest.

#### Pagers

To deliver the random signals for questionnaire completion to the athletes, Motorola (Schaumberg, Illinois, USA; model: PageOne Minicall) pagers were used. To rule out the possibility of accidental errors in dialling the pager numbers, calls were performed by means of a personal computer and a modem using the AvantPager 32 (version 4.00) software.

### Procedures

This study was approved by the Ethics Committee of the local university. During an initial interview, participants were briefed about the aims and procedures of the study, informed consent was obtained and anonymity and confidentiality was assured. They then completed the DQ, the SCAT, performance expectations item and the neuroticism and extraversion scales of the NEO PI-R. Participants were given a pager and were well familiarized with its use. They were also given a booklet containing the DES-IV, items assessing type, controllability, importance and desirability of concerns and an item measuring performance appraisal at the competition. Each booklet included enough experience sampling questionnaires to last for the entire period of sampling. Participants were paged five random times a day over a period of seven consecutive days before and three consecutive days after the competition. The day was divided into five blocks between the hours of 9:00 and 21:30. Within each of these periods one randomized pager signal was sent with a minimum of 30-min delay between the signals. Upon reception of the signal, participants completed the experience sampling questionnaires. They first indicated the date and time of the day of completion. Second, they rated their momentary affective states on the DES-IV. Finally, they reported the type, pleasantness/desirability, controllability and importance of eventual concern experienced in the interval since their last report. Participants were instructed that if the pager was accidentally turned off or malfunctioned, or if they were unable to answer within 30 min of the signal, they should not complete the questionnaires for that sampling (Cerin & Barnett, 2006). On the day of the competition, the participants completed the usual set of questionnaires approximately 1 h before and immediately after the competition. They also appraised their performance. An inconvenience allowance of £35 was given to the participants that completed the study. All athletes had a 3–6-week interval between the last day of the study and the next competition.

Compliance with the procedures was very good. Participants completed an average of 93.5% of all possible responses within the time limit, for an average of 48.6 out of 52 valid responses per participant. The average time delay between the signal from the pager and the actual completion of the questionnaires was 8.0 min (SD=8.7). Compliance rate was unrelated to age, sport experience, competitive trait anxiety, extraversion, neuroticism and day of the study.

### Data analysis

Data on controllability and importance of reported concerns were aggregated to provide a single estimate for each participant per type of concern. *t*-tests for dependent samples were used to examine the difference in perceived importance and controllability of competition-related and competition-extraneous concerns.

This study adopted a mixed nomothetic–idiographic approach. Consequently, principal components analyses of the responses on 11 subscales of the DES-IV were conducted on both mean scores aggregated per subject (nomothetic data) and within-subject *z-*scores (idiographic data). These analyses provided a criterion for grouping affects into sets of outcomes for subsequent multivariate, multilevel regression models (Snijders & Bosker, 1999) that examined the associations of athletes' cognitive appraisal and personality traits with pre- and post-competition affects.

Multilevel linear models are a variant of the multiple regression models which are appropriate for datasets with a multilevel (hierarchical) structure. They are particularly useful for the analysis of longitudinal, ESM data, allowing for missing observations and observations unequally spaced in time (Cerin, 2004). The term “multivariate” refers to the presence of two or more dependent variables or criteria. Specifically, it refers to the fact that athletes' pre-competition affective states were assessed on 11 discrete affects grouped into three sets of inter-correlated affects, following the results of principal components analyses. These were hostility (disgust and anger), positive affects (surprise, enjoyment and interest) and negative affects (shyness, shame, sadness, fear, self-hostility and guilt).

A multivariate multilevel linear model was constructed for each set of pre- and post-competition affects. The dataset comprised one or more daily observations on three groups of dependent variables (criteria) nested within days within subjects. These four levels (sources of variation) are referred to as intra-day, day, person and criteria levels. The main effects models encompassed five predictors at the intra-day level, one predictor at the day level and three predictors at the person level. The intra-day level predictors were presence of concern (yes/no), concern context (competition-extraneous vs competition-related), desirability (desirable vs undesirable), controllability and importance of the reported concern. Temporal proximity to competition (day of study) represented the only predictor at the day level. Expected performance (pre-competition) or performance appraisals (post-competition), competitive trait anxiety and neuroticism were included in the equation as person-level predictors of hostility and negative affects. Neuroticism was replaced with extraversion in the positive affects models.

The predictor “presence of concern” indicated whether a subjectively significant event or thought had been reported and was aimed to help differentiate pre-competition mood (affective state that has no apparent triggering stimulus) from pre-competition emotions (affective states caused by an identifiable event/cognition). Concern context indicated whether the reported concern was explicitly related to the forthcoming competition and was included to analyze the difference in athletes' emotions triggered by competition-related and competition-extraneous concerns. Concern controllability (by the self) and importance were included as dimensions of coping potential/self-accountability and goal relevance determining athletes' affective reaction. Concern desirability was added to account for goal congruence.

Appropriate interaction terms were added to the regression models described above to examine the moderating effects of personality on the relationships of temporal proximity to competition, concern context and performance appraisals with affective states. A Proximity to Competition by Concern Context interaction term was also added to the models to examine whether the influence of competition-related concerns on athletes' psychological state would depend on the temporal proximity to the competition.

All continuous predictors were standardized (mean 0, variance 1) and the variable “day to or from competition” was centered and assumed values from −3.5 to 3.5 for the models of pre-competition affects, and values from −1.5 to 1.5 for the models of post-competition affects (Snijders & Bosker, 1999). The variable presence of concern was dummy-coded as 1 or 0. Effect coding was used for the categorical variables of “concern context” (1 if competition-related concern, −1 if competition-extraneous concern, 0 if no concern) and concern desirability (1 if desirable concern, −1 if undesirable concern, 0 if no concern) so that the estimated effect of presence of concern would not change after their inclusion. The concern controllability and importance were assigned the value zero if no source of concern was reported.

A hundred and thirty-one observations with missing data on any of the predictors were deleted. This resulted in a total of 1897 valid observations. Significance of the regression coefficients was established by dividing the estimated effect by its standard error. This ratio is approximately normally distributed (Snijders & Bosker, 1999). The likelihood ratio test was used to test the significance of autocorrelation and residual variances at each level. The amount of variance in affects explained by the models was established by calculating the proportional reduction of error (*R*^2^) using the method described by Snijders and Bosker (1999). Testing of significance of regression coefficients was two-tailed, while that of autocorrelation and variances was one-tailed (Snijders & Bosker, 1999). An α level of 0.05 was adopted.

## Results

On average, a greater importance was attributed to competition-related (6.02±0.89) than competition-extraneous concerns [5.07±1.00, *t*(38)=5.51; *P*<0.01]. Athletes perceived having greater control over competition-related (4.47±1.09) than competition-extraneous concerns [3.64±1.74, *t*(38)=2.53; *P*<0.05].

### Models of pre- and post-competition affects

The examined multivariate, multilevel models of affects explained from 4% to 44% of the variance pre-competition and 13–56% of the variance post-competition (Tables 1–4). Overall, athletes' mood (defined as the level of affects in absence of a concern and quantified by intercept values of models in Tables 1–4) was characterized by low levels of negative affects and moderate levels of positive affects. While, pre-competition, “fear” was the subscale with the highest average score in absence of concerns (3.49±0.08), sadness, anger and guilt were the subscales with the highest scores post-competition.

**Table 1:** Regression coefficients and their standard errors (in brackets) for pre-competition hostility and negative affects

Predictor	Disgust	Anger	Guilt	Shyness	Self-hostility	Shame	Sadness	Fear
Intercept	3.11 (0.04)^c^	3.11 (0.08)^c^	3.20 (0.07)^c^	3.18 (0.06)^c^	3.11 (0.04)^c^	3.20 (0.08)^c^	3.27 (0.07)^c^	3.49 (0.08)^c^
Intra-day level								
Presence of concern	0.13 (0.04)^b^	0.91 (0.08)^c^	0.26 (0.04)^c^	0.17 (0.04)^c^	0.31 (0.04)^c^	0.12 (0.03)^c^	0.27 (0.05)^c^	0.52 (0.05)^c^
Concern context	0.03 (0.04)	−0.05 (0.06)	0.01 (0.04)	0.04 (0.03)	0.00 (0.03)	0.05 (0.03)	0.01 (0.04)	0.32 (0.04)^c^
Desirability	−0.13 (0.04)^b^	−0.84 (0.07)^c^	−0.30 (0.04)^c^	−0.11 (0.03)^c^	−0.28 (0.03)^c^	−0.02 (0.03)	−0.34 (0.04)^c^	−0.24 (0.05)^c^
Controllability	−0.01 (0.04)	−0.13 (0.06)^a^	0.10 (0.04)^b^	0.00 (0.03)	0.02 (0.03)	−0.04 (0.02)^a^	−0.05 (0.04)	−0.08 (0.04)^a^
Importance	−0.06 (0.04)	0.12 (0.05)^a^	0.05 (0.03)	0.03 (0.03)	0.06 (0.03)^a^	0.01 (0.03)	0.00 (0.03)	0.05 (0.05)
Day level								
Day	0.00 (0.01)	0.04 (0.02)	−0.02 (0.01)	0.01 (0.01)	0.03 (0.01)^a^	0.01 (0.01)	0.01 (0.01)	0.23 (0.03)^c^
Person level								
Expected performance	−0.01 (0.04)	−0.14 (0.08)	−0.07 (0.07)	−0.07 (0.05)	−0.09 (0.04)^a^	−0.13 (0.07)	−0.13 (0.08)	−0.15 (0.08)
CTA	0.07 (0.05)	−0.10 (0.09)	0.06 (0.08)	0.05 (0.07)	0.02 (0.05)	0.04 (0.10)	0.04 (0.09)	−0.01 (0.10)
Neuroticism	0.02 (0.05)	0.19 (0.09)^a^	0.04 (0.08)	0.07 (0.08)	0.05 (0.05)	0.09 (0.10)	0.18 (0.09)^a^	0.18 (0.10)
Interaction terms								
CTA by day	0.01 (0.01)	0.03 (0.02)	0.01 (0.02)	0.00 (0.01)	0.02 (0.01)	−0.01 (0.01)	0.03 (0.02)	0.01 (0.03)
Neuroticism by day	−0.02 (0.05)	−0.05 (0.05)	0.01 (0.02)	0.00 (0.01)	−0.02 (0.01)	0.01 (0.01)	−0.02 (0.02)	0.07 (0.03)^a^
CTA by context	0.02 (0.03)	0.11 (0.05)^a^	0.05 (0.03)	0.08 (0.03)^b^	0.06 (0.03)^a^	0.07 (0.03)^b^	0.02 (0.03)	0.14 (0.04)^c^
Day by context	0.01 (0.02)	−0.04 (0.03)	0.04 (0.02)^a^	0.00 (0.01)	0.00 (0.01)	0.00 (0.01)	0.02 (0.02)	0.15 (0.02)^c^
Variance terms								
Person level	0.03^a^	0.15^b^	0.14^b^	0.11^b^	0.05^a^	0.21^b^	0.17^b^	0.12^b^
Day level	0.05^b^	0.15^c^	0.11^b^	0.07^b^	0.05^b^	0.02^a^	0.13^c^	0.59^c^
Intra-day level	0.46^c^	1.25^c^	0.45^c^	0.31^c^	0.37^c^	0.30^c^	0.47^c^	0.52^c^
*R*^2^	0.04	0.26	0.10	0.04	0.13	0.06	0.17	0.44

a *P*<0.05;

b *P*<0.01;

c *P*<0.001.

CTA, competitive trait anxiety; Day, day to competition; Presence of concern, yes (1) vs no (0); Concern context, competition-related (1) vs competition extraneous (−1); Desirability, desirable concern (1) vs undesirable concern (−1). All continuous predictors standardized.

**Table 2:** Regression coefficients and their standard errors (in brackets) for pre-competition positive affects

*Predictor*	Enjoyment	Surprise	Interest
Intercept	7.29 (0.27)^c^	4.30 (0.17)^c^	5.90 (0.21)^c^
Intra-day level			
Presence of concern	−0.09 (0.11)	0.59 (0.08)^c^	1.08 (0.12)^c^
Concern context	0.09 (0.09)	0.06 (0.07)	0.43 (0.11)^c^
Pleasantness	1.40 (0.10)^c^	0.61 (0.07)^c^	1.10 (0.11)^c^
Controllability	0.04 (0.09)	−0.23 (0.06)^c^	0.06 (0.10)
Importance	0.03 (0.08)	−0.10 (0.06)	0.18 (0.09)^a^
Day level			
Day	−0.14 (0.05)^b^	0.03 (0.04)	−0.05 (0.05)
Person level			
Expected performance	0.67 (0.27)^a^	0.35 (0.17)^a^	0.45 (0.22)^a^
CTA	0.02 (0.26)	−0.08 (0.17)	−0.05 (0.22)
Extraversion	0.59 (0.26)^a^	0.08 (0.16)	0.10 (0.22)
Interaction terms			
CTA by day	−0.05 (0.05)	−0.03 (0.04)	−0.02 (0.05)
Extraversion by day	0.04 (0.05)	0.01 (0.04)	0.04 (0.06)
CTA by context	−0.04 (0.08)	−0.03 (0.04)	−0.02 (0.09)
Day by context	−0.05 (0.04)	0.04 (0.03)	0.07 (0.04)
Variance terms			
Person level	2.50^c^	0.99^b^	1.49^c^
Day level	0.62^c^	0.13^b^	0.49^c^
Intra-day level	2.73^c^	1.61^c^	3.37^c^
*R*^2^	0.26	0.18	0.26

a *P*<0.05;

b *P*<0.01;

c *P*<0.001.

CTA, competitive trait anxiety; Day, day to competition; Presence of concern, yes (1) vs no (0); Concern context, competition-related (1) vs competition extraneous (−1); Desirability, desirable concern (1) vs undesirable concern (−1). All continuous predictors standardized.

**Table 3:** Regression coefficients and their standard errors (in brackets) for post-competition hostility and negative affects

Predictor	Disgust	Anger	Guilt	Shyness	Self-hostility	Shame	Sadness	Fear
Intercept	3.16 (0.09)^c^	3.51 (0.13)^c^	3.49 (0.09)^c^	3.31 (0.09)^c^	3.31 (0.09)^c^	3.22 (0.07)^c^	3.63 (0.12)^c^	3.18 (0.07)^c^
Intra-day level								
Presence of concern	0.33 (0.08)^c^	0.97 (0.12)^c^	0.72 (0.09)^c^	0.33 (0.08)^c^	0.69 (0.08)^c^	0.33 (0.06)^c^	0.59 (0.10)^c^	0.25 (0.05)^c^
Concern context	0.02 (0.08)	−0.15 (0.11)	0.34 (0.09)^c^	0.13 (0.07)	0.42 (0.08)^c^	0.13 (0.05)^b^	0.20 (0.09)^a^	0.00 (0.05)
Pleasantness	−0.37 (0.06)^c^	−1.12 (0.10)^c^	−0.45 (0.08)^c^	−0.15 (0.06)^a^	−0.36 (0.07)^c^	−0.11 (0.05)^a^	−0.76 (0.08)^c^	−0.22 (0.04)^c^
Controllability	−0.18 (0.06)^b^	−0.13 (0.09)^a^	0.05 (0.07)	−0.11 (0.06)	−0.05 (0.06)	−0.04 (0.05)	−0.19 (0.07)^a^	−0.05 (0.04)
Importance	0.05 (0.06)	0.36 (0.10)^c^	0.32 (0.07)^c^	0.10 (0.07)	0.16 (0.07)^a^	0.05 (0.05)	0.33 (0.08)^c^	0.15 (0.04)^c^
Day level								
Day	−0.15 (0.07)^a^	−0.46 (0.10)^a^	−0.31 (0.10)^c^	−0.13 (0.06)^a^	−0.25 (0.08)^b^	−0.13 (0.04)^b^	−0.41 (0.10)^c^	−0.13 (0.05)^a^
Person level								
Performance appraisal	0.06 (0.14)	−0.29 (0.18)	−0.36 (0.18)^a^	−0.22 (0.13)	−0.26 (0.13)^a^	−0.24 (0.10)^a^	−0.41 (0.18)^a^	−0.22 (0.11)^a^
CTA	0.06 (0.14)	0.15 (0.14)	0.23 (0.15)	0.14 (0.10)	0.20 (0.10)^a^	0.07 (0.08)	0.14 (0.14)	0.06 (0.09)
Neuroticism	0.01 (0.11)	0.06 (0.14)	0.14 (0.15)	0.06 (0.10)	0.05 (0.11)	0.07 (0.08)	0.12 (0.14)	0.05 (0.09)
Interaction terms								
CTA by day	−0.04 (0.08)	−0.18 (0.11)	−0.24 (0.10)^a^	−0.12 (0.07)	−0.26 (0.09)^b^	−0.13 (0.05)^b^	−0.28 (0.11)^b^	−0.10 (0.06)
Neuroticism by day	0.02 (0.08)	0.03 (0.11)	−0.08 (0.10)	−0.06 (0.06)	0.00 (0.10)	−0.02 (0.05)	−0.03 (0.11)	−0.06 (0.06)
CTA by performance	0.13 (0.11)	−0.16 (0.14)	−0.32 (0.13)^a^	−0.20 (0.09)^a^	−0.09 (0.10)	−0.13 (0.07)	−0.23 (0.12)	−0.12 (0.08)
Neuroticism by performance	−0.12 (0.14)	0.01 (0.19)	0.06 (0.17)	−0.08 (0.12)	0.01 (0.13)	−0.10 (0.10)	−0.12 (0.17)	−0.11 (0.10)
Day by context	−0.12 (0.14)	−0.10 (0.12)	−0.18 (0.09)	−0.01 (0.08)	−0.21 (0.08)^b^	0.00 (0.06)	0.13 (0.02)	−0.05 (0.05)
Variance terms								
Person level	0.09	0.15^b^	0.11	0.09^a^	0.00	0.06	0.06	0.08^a^
Day level	0.59^c^	0.90^c^	0.95^c^	0.28^c^	0.77^c^	1.07^c^	0.28^c^	0.15^b^
Intra-day level	0.35^c^	0.90^c^	0.53^c^	0.57^c^	0.43^c^	0.59^c^	0.17^c^	0.27^c^
*R*^2^	0.13	0.47	0.53	0.34	0.52	0.56	0.27	0.40

a *P*<0.05;

b *P*<0.01;

c *P*<0.001.

CTA, competitive trait anxiety; Day, day to competition; Presence of concern, yes (1) vs no (0); Concern context, competition-related (1) vs competition extraneous (−1); Desirability, desirable concern (1) vs undesirable concern (−1). All continuous predictors standardized.

**Table 4:** Regression coefficients and their standard errors (in brackets) for post-competition positive affects

Predictor	Enjoyment	Surprise	Interest
Intercept	7.29 (0.28)^c^	4.31 (0.21)^c^	5.94 (0.26)^c^
Intra-day level			
Concern	−0.19 (0.17)	0.68 (0.14)^c^	0.77 (0.20)^c^
Context	0.30 (0.13)^a^	0.18 (0.16)	0.28 (0.18)
Pleasantness	1.80 (0.15)^c^	1.04 (0.12)^c^	1.74 (0.16)^c^
Controllability	0.21 (0.14)	−0.46 (0.11)^c^	0.04 (0.16)
Importance	0.05 (0.15)	0.18 (0.12)	0.28 (0.17)
Day level			
Day	−0.01 (0.13)	0.07 (0.10)	0.06 (0.13)
Person level			
Performance appraisal	1.41 (0.43)^b^	0.82 (0.32)^a^	0.95 (0.40)^a^
CTA	−0.29 (0.34)	0.29 (0.26)	0.02 (0.32)
Extraversion	0.27 (0.31)	0.03 (0.23)	0.14 (0.28)
Interaction terms			
CTA by day	0.31 (0.14)^a^	0.19 (0.10)	0.04 (0.14)
Extraversion by day	0.04 (0.13)	0.10 (0.10)	−0.04 (0.17)
CTA by performance	0.00 (0.32)	−0.25 (0.24)	−0.27 (0.30)
Day by context	−0.23 (0.17)	−0.47 (0.14)^c^	−0.23 (0.17)
Variance terms			
Person level	2.21^c^	1.21^c^	1.86^c^
Day level	1.09^c^	0.46^c^	0.76^c^
Intra-day level	2.17^c^	1.43^c^	2.99^c^
*R*^2^	0.39	0.30	0.31

a *P*<0.05;

b *P*<0.01;

c *P*<0.001.

CTA, competitive trait anxiety; Day, day to competition; Presence of concern, yes (1) vs no (0); Concern context, competition-related (1) vs competition extraneous (−1); Desirability, desirable concern (1) vs undesirable concern (−1). All continuous predictors standardized.

#### Appraisal dimensions

Significant positive associations with concern importance were observed for pre- and post-competition anger and self-hostility, pre-competition interest and post-competition guilt, sadness and fear (Tables 1–4). For most of the remaining affects, albeit not statistically significant, the effect of importance of a concern was in the predicted direction. Concern desirability (goal congruence) was consistently negatively associated with negative affects (Tables 1 and 3) and positively associated with positive affects (Tables 2 and 4). Perceived personal control (coping potential) tended to be negatively related to the subscales of anger, shame, fear, surprise, sadness and disgust (Tables 1–4) and positively related to guilt (Tables 1 and 3). While the associations between controllability of a source of concern and the enjoyment, self-hostility and interest subscales were in the expected direction, they were not statistically reliable.

In the pre-competition period, reporting of competition-related concerns was associated with higher scores on fear (Table 1) and interest (Table 2) than was reporting of competition-extraneous concerns. After the contest, competition-related concerns were predictive of higher scores on guilt, self-hostility, shame, sadness and enjoyment (outcome-dependent affects). Performance expectations (future expectations) tended to show weak but non-significant negative associations with outcome-dependent negative affects and fear (Table 1) and positive, significant association with positive affects (Table 2). Performance appraisal (goal congruence) was significantly negatively related to fear and most post-competition outcome-dependent negative affects (guilt, self-hostility and sadness; Table 3), and positively related to positive affects (Table 4). Average performance appraisal was 5.46 (SD=1.64), indicating that athletes on average appraised their performance to be slightly higher than their average standard.

#### Concern context by proximity of competition interaction effects

In the pre-competition period, proximity of competition interacted with concern context only in the case of guilt and fear (Table 1). An analysis of these interactions revealed that while competition-extraneous concerns were associated with relatively stable low scores on guilt and fear throughout the whole pre-competition period, competition-related concerns were associated with increasingly higher scores on fear and guilt as the competition approached. Post-competition, interactive effects of concern context and time from competition were observed for surprise and self-hostility (Tables 3 and 4). The difference between competition-related and competition-extraneous concerns in post-competition levels of these affects decreased with time.

#### Personality traits

Neuroticism was independently associated with pre-competition anger and sadness (Table 1), and extraversion with pre-competition enjoyment (Table 2). Athletes with higher levels of trait anxiety reported higher levels of pre-competition fear, shyness, self-hostility and shame for competition-related than for competition-extraneous concerns (Table 1). In contrast, among low-anxious athletes, fear was the only affect to be higher for competition-related than competition-extraneous concerns (although to a lesser extent than in their more anxious counterparts). A significant neuroticism by Proximity of Competition interaction effect on pre-competition fear was observed. Athletes higher in neuroticism experienced a steeper increase in fear as the competition approached than athletes with lower neuroticism. Five significant Competitive Trait Anxiety by Proximity of Competition interaction effects on post-competition affects were found. Specifically, while levels of post-competition guilt, self-hostility, shame and sadness were low and stable across time in low-anxious athletes, they were elevated immediately after the competition in high-anxious athletes and decreased thereafter (Table 3). The opposite was observed for post-competition enjoyment (Table 4).

Finally, no significant interactions of neuroticism and performance appraisals at the competition on post-competition affects were found. However, the effect of performance appraisals on post-competition affects was moderated by competitive trait anxiety (Table 3). Performance appraisals did not impact on post-competition guilt and shyness of low-anxious athletes. However, they were negatively associated with post-competition guilt and shyness in high-anxious athletes.

## Discussion

The present study is a mixed nomothetic–idiographic process analysis of athletes' affective states arising from both competition-related and competition-extraneous concerns. This type of approach can (1) provide a better view of the importance of an athletic contest in relation to other sources of concern; (2) identify key cognitive determinants influencing affective states; and (3) identify personal and situational factors influencing the relationships between cognition and affects.

### Comparing reactions to competition-related and competition-extraneous concerns

This study confirmed that, in general, athletes perceived the competition as a challenging, positive and important event (Graham et al., 2002; Cerin, 2003;). Within the examined study timeframe, athletes attributed more importance to competition-related than competition-extraneous events or cognitions. Moreover, as reported in a companion paper (Cerin & Barnett, 2006), athletes' affective experience was typified by moderate levels of positive and low levels of negative affects throughout the whole period of testing, and was accompanied by relatively frequent reports of desirable competition-related concerns.

Before the contest, competition-related concerns triggered higher levels of fear and interest than did competition-extraneous concerns. Additionally, while intensity of fear evoked by competition-extraneous concerns did not change across time, competition-related concerns yielded increasingly higher levels of fear as the competition approached. This indicates that, in the athletes' mind, the competition was both a threatening and challenging event necessitating increasingly more attention and energy mobilization than other types of concern (Cerin, 2003). After the contest, competition-related concerns were associated with higher levels of outcome-dependent negative (e.g., sadness and guilt) and positive emotions (enjoyment), highlighting again the relatively higher importance given to the contest as compared with other events. However, this difference in affective reactions was transitory, and similarly to Nicholls et al. (2009) study, dissipated in 2 days. In this regard, it is known that individuals usually adapt very rapidly to positive and negative changes in life circumstances and events (Diener et al., 1999). According to the theory of dynamic equilibrium, levels of affects are in the main determined by the personality traits of neuroticism and extraversion (Headey, 2006). While life events can impact on affective states, their levels tend to return to their set equilibrium point relatively quickly. Recent longitudinal studies suggest this to be particularly true for individuals high in extraversion, as were the participants in this study (Headey, 2006).

Differences between competition-related and competition-extraneous concerns were also found in perceived (personal) controllability of concerns, with competition-related episodes being perceived to be more controllable than competition-extraneous events. This is understandable as highly skilled athletes (as in this study) are likely to have high levels of perceived competence in their sport, which is usually defined as the perceived personal control over a situation (Harter, 1981).

### Appraisal dimensions and affective states

One of the aims of this study was to simultaneously examine the inter- and intra-individual relationships between primary and secondary appraisal dimensions and affective states. The hypothesis that perceived importance of a concern would be associated with intensity of affects was only partially supported. It is possible that this might due to asking participants to report only events that triggered an emotional reaction. Such procedure results in the omission of unimportant events or cognitions that had no impact on an individual's affective state, thereby yielding an overly restricted range of values for concern importance. In this study, the mean reported importance was 5.53, with a standard deviation of 0.72 on a seven-point scale, confirming our supposition. However, it should be noted that this particular measure of sources of concern was used in order to identify concerns *causing* a particular affective state and differentiating mood from emotions. Notably, inconsistent relations between goal importance and post-competition emotions, attributable to restricted variability of the independent variable, were also found in a cross-sectional study (Graham et al., 2002). Similarly to this study, for unknown reasons, stronger relationships were observed between event importance and negative, rather than positive affects.

As hypothesized, goal congruence, measured in the form of desirability of an event or cognition and performance appraisals, was consistently negatively associated with negative affects and positively associated with positive affects. This is in line with earlier studies that reported goal congruence to be the appraisal dimension most robustly associated with affective states in sport (Graham et al., 2002; Uphill & Jones, 2007;). Also, as hypothesized, personal control over a source of concern was positively associated with guilt and negatively associated with anger, shame, fear, surprise, and sadness (Lazarus, 1999; Thompson et al., 2004;). Although seemingly counterintuitive, a positive association between personal control and guilt was expected because guilt is, by definition, an emotional reaction associated with specific, controllable behaviors that violate the individual's internal standards, resulting in a state of remorse and regret (Lazarus, 1999). Finally, future expectations about performance were significantly positively related to pre-competition positive affects but only marginally negatively related to the negative affects of self-hostility (*P*<0.05), sadness (*P*=0.09) and fear (*P*=0.07). This suggests that the prospect of performing poorly at a competition was perceived as a threatening situation (deducible from the association with fear) that could not be improved (deducible from the association with sadness) for which the athletes felt responsible (deductible from the association with self-hostility).

In conclusion, in line with the theoretical model of Smith and Lazarus (1993), this study provides support for the supposition that the primary appraisal dimension of goal congruence (in the form of event desirability), and the secondary appraisal dimensions of coping potential (in the form of perceived personal control) and future expectation (in the form of expected performance) are important antecedents of athletes' affective states in temporal proximity of a competition.

### Personality traits

In line with personality theory (Costa & McCrae, 1992) and previous findings in the sport literature (Cerin, 2004), extraversion was positively related to pre-competition enjoyment, and neuroticism was associated with pre-competition sadness and anger. However, no significant *independent* effects of these two personality traits were found post-competition. Also, contrary to expectations, no significant independent associations between neuroticism and pre-competition fear, shame, shyness, disgust, self-hostility and guilt emerged. These results were likely due to the effects of extraversion and neuroticism on affective states being mostly mediated by cognitive appraisal. In other words, it is postulated that these two personality traits determine how a situation is appraised, which, in turn, determines one's affective state (Costa & McCrae, 1992). If this is true then the effects of neuroticism and extraversion on affective states should be close to nil when controlling for appraisal, while they should be statistically significant when not controlling for appraisal dimensions. However, although interesting, an analysis of the effects of personality traits mediated by appraisal was outside the scope of this paper.

Personality traits interacted with competition proximity and dimensions of appraisal to influence athletes' affective states. Athletes higher in neuroticism tended to experience greater increases in pre-competition fear than their counterparts. This is in contrast to a study by Prapavessis and Grove (1994) who reported no significant interaction effects of neuroticism and competition proximity on pre-competition tension. However, their study used a different measure of fear-like affect; monitored athletes for only 2 days; and examined a sport that is associated with a low level of arousal (rifle shooting). Thus, their study might have been underpowered to detect differences in temporal changes of pre-competition tension.

Competitive trait anxiety was an effect modifier of post-competition temporal patterns for five different affects. The discrepancy in “significance” of effects might be due to competitive trait anxiety being a domain-specific and neuroticism being a generic personality trait. An examination of these interactions revealed that high-anxious athletes experienced higher levels of negative affects and lower levels of positive affects immediately after the competition, which gradually dissipated in the next 3 days. In contrast, low-anxious athletes exhibited stable positive patterns of post-competition affects. These results confirm previous research which demonstrated that athletes with high levels of competitive trait anxiety are more reactive to failure and social evaluation and experience greater shame and upset in the event of poor performance (Rainey & Cunningham, 1988).

That individuals with high levels of competitive trait anxiety are more vulnerable to sport-related evaluative situations was confirmed further by them reacting to poor perceived performance with heightened levels of shyness and guilt, while their low-anxious counterparts appeared not to be reactive to performance appraisals. Also, athletes with high levels of competitive trait anxiety experienced greater differences in pre-competition fear, shame, shyness and self-hostility between competition-related and competition-extraneous concerns than did their counterparts. Considering the relational meaning of these affects, this suggests that anxious athletes tended to associate the competitive events with thoughts or feelings of ego vulnerability (shyness), disappointment in the self and self-blame for an eventual failure (shame and self-hostility) to greater extent than did low-anxious athletes (Izard, 1991; Lazarus, 1999;). These findings indicate that high-anxious individuals do not get as much satisfaction or enjoyment out of a competitive event as do individuals low on this personality trait. This is consonant with previous published research (Rainey & Cunningham, 1988).

### Mood vs emotions

As noted earlier, by asking participants to report affective states and concerns associated with them (if any), this study attempted to differentiate between emotions and mood. Thus, the intercepts of the regression models of affects are to be interpreted as the average mood experienced halfway through the pre- or post-competition period for the average subject. Notably, fear was the strongest negative “mood” pre-competition, while guilt, sadness and anger were the strongest “moods” post-competition. Also, the regression coefficients of proximity of competition represent estimates of the linear increase or decrease in mood across time. It is interesting that, for the average participant, levels of self-hostility, fear and enjoyment in absence of identifiable causes showed a significant linear trend as the competition approached. While the first two affects increased, enjoyment decreased with time. Also, a post-competition decreasing linear trend was observed in all negative affects, while an increasing trend was observed for post-competition enjoyment. This suggests that, in the examined timeframe, the competition exerted a generalized negative impact on athletes' mental well-being, of which they may have not been aware. In this regard, research in organizational and clinical psychology has reported a certain degree of emotional “permeability” or mutual influence between life domains (Heller & Watson, 2005). This is thought to be due to the limited psychological and physiological resources that can be allocated between domains and to the relative persistence of affective experience emerging from events relevant to the individual (Heller & Watson, 2005). This study suggests that emotional permeability may occur between competitive sport activities and other life domains. This issue is presented in a companion paper (Cerin & Barnett, unpublished observation).

### Study limitations

Our study is limited by its sample size, gender and type of sport examined. Methodologically, it is limited because it allowed participants to report only one concern per assessment, assessed concern desirability dichotomously, used a unidimensional measure of competitive anxiety rather than also assessing trait anxiety direction, and did not examine the impact of the primary appraisal of ego involvement on emotions. The last limitation was due to the intensive nature of the study, requiring the participants to be assessed on affects and their correlates multiple times a day for 10 consecutive days. To ensure sufficient compliance and minimize attrition and selection bias, it was important that completion of the questionnaire be easy and quick (Scollon et al., 2003). The assessment of ego involvement would have required the participants to either provide a time-consuming written elaboration of the goal at stake or engage in a rather cognitively demanding task of type-of-goal identification. Although the longitudinal nature of this study provides stronger evidence of a causal relationship between appraisal and affective states than do cross-sectional studies, causal relationships cannot be inferred due to the lack of experimental manipulation of independent variables.

Apart from addressing the above limitations, to gain a clearer picture of events and situations influencing pre- and post-competition affects, future research needs to examine in greater detail the prevalence, temporal patterns, and effects of types of competition-extraneous (e.g., work, family and social network stressors) and competition-related (e.g., stressors related to training practices, social interaction with teammates and environmental conditions) concerns. It also needs to examine the role of coping mechanisms (Gaudreau et al., 2002; Nicholls et al., 2009;), a key variable that follows and shapes emotions (Lazarus, 1999) which can provide crucial information on effective strategies for the regulation of affective states. To maximize the practical value of such research, future studies need to identify situational and personal determinants of individual differences in appraisal–emotion relationships and of pre- and post-competition affective phenomena in general.

### Practical implications

From a practical standpoint, this study highlights the need for sport psychologists to identify and assist the emotional regulation of athletes that are high in neuroticism and competitive trait anxiety. This is because these traits are predictive of dysfunctional reactions to competition that may hinder performance and athletes' mental well-being. Specifically, in this study, neuroticism was predictive of heightened pre-competition sadness, which has been consistently related to impaired performance (e.g., Lane & Terry, 2000; Cerin, 2003;), likely due to its deactivating effects on behavior (Izard, 1991; Lazarus, 1999;). Competitive trait anxiety was associated with increased competition-related shame and shyness, emotions that elicit submissive and avoidance behavior, and self-focused attention (Izard, 1991), which are bound to negatively affect performance, especially in open-skilled combat sports. Competitive trait anxiety was also predictive of dysfunctional adaptation after the competition, particularly after poor performance.

## Perspectives

By using a mixed nomothetic–idiographic framework this study identified some cognitive, situational and personal factors associated with male martial artists' affective responses in the week preceding and 3 days following a major competition. The competition was the most important and stressful event that the athletes experienced in the examined period. Support was found for primary (goal congruence) and secondary (future expectations and coping potential) appraisals as determinants of athletes' affects. It is unclear whether the inconsistent associations between goal relevance and affects observed are a measurement artefact or a deficiency in the proposed theoretical model. This study highlighted the importance of competitive trait anxiety as both a moderator of the relationships between appraisal and affective states and a determinant of affective states, while neuroticism and extraversion were identified as determinants only. Sport psychologists need to identify athletes that are high in competitive trait anxiety and neuroticism, help them develop a more positive attitude toward competition and reduce negative affects that may be detrimental to performance. Further research is needed to clarify the relationships of competition-extraneous and competition-related sources of stress and athletes' affective states. It is particularly important to identify factors determining individual differences in such relationships.

## References

[b1] Cerin E (2003). Anxiety versus fundamental emotions as predictors of perceived functionality of pre-competitive emotional states, threat, and challenge in individual sports. J Appl Sport Psychol.

[b2] Cerin E (2004). Predictors of competitive anxiety direction in male Tae Kwon Do practitioners: a multilevel mixed idiographic/nomothetic interactional approach. Psychol Sport Exerc.

[b3] Cerin E, Barnett A (2006). A processual analysis of basic emotions and sources of concern as they are lived before and after a competition. Psychol Sport Exerc.

[b4] Cerin E, Szabo A, Hunt N, Williams C (2000). Temporal patterning of competitive emotions: a critical review. J Sports Sci.

[b5] Cerin E, Szabo A, Williams C (2001). Is the experience sampling method (ESM) appropriate for studying pre-competitive emotions?. Psychol Sport Exerc.

[b6] Costa PT, McCrae RR (1992). NEO PI-R: professional manual.

[b7] Diener E, Suh EH, Lucas RE, Smith HL (1999). Subjective well-being: three decades of progress. Psychol Bull.

[b8] Ekman P, Ekman P, Davidson RJ (1994). Moods, emotions, and traits. The naure of emotion: fundamental questions.

[b9] Gaudreau P, Blondin JP, Lapierre AM (2002). Athletes' coping during competition: relationship of coping strategies with positive affect, negative affect, and performance-goal discrepancy. Psychol Sport Exerc.

[b10] Graham TR, Kowalski KC, Crocker PRE (2002). The contributions of goal characteristics and causal attributions to emotional experience in youth sport participants. Psychol Sport Exerc.

[b11] Gross JJ (2006). Handbook of emotion regulation.

[b12] Hanin YL, Hanin YL (2000). Introduction: an individualized approach to emotion in sport. Emotions in sport.

[b13] Hanton S, Jones G (1997). Antecedents of intensity and direction dimensions of competitive anxiety as a function of skill. Psychol Rep.

[b14] Harter S, Roberts GC, Landers DM (1981). The development of competence motivation in the mastery of cognitive and physical skills: is there a place for joy?. Psychology of motor behavior and sport – 1980.

[b15] Hassmén P, Koivula N, Hansson T (1998). Precompetitive mood states and performance in elite male golfers: do trait characteristics make a difference?. Percept Mot Skills.

[b16] Headey B (2006). Subjective well-being: revisions to dynamic equilibrium theory using national panel data and panel regression methods. Soc Indic Res.

[b17] Heller D, Watson D (2005). The dynamic spillover of satisfaction between work and marriage: the role of time and mood. J Appl Psychol.

[b18] Hormuth SE (1986). The sampling of experiences in situ. J Pers.

[b19] Huband ED, McKelvie JS (1986). Pre and post game state anxiety in team athletes high and low in competitive trait anxiety. Int J Sport Psychol.

[b20] Izard CE (1991). The psychology of emotions.

[b21] Izard CE, Libero DZ, Putam P, Haynes OM (1993). Stability of emotion experiences and their relation to traits of personality. J Pers Soc Psychol.

[b22] Lane AM, Terry PC (2000). The nature of mood: development of a conceptual model with a focus on depression. J Appl Sport Psychol.

[b23] Lazarus RS (1999). Stress and emotion: a new synthesis.

[b24] Martens R, Vealey RS, Burton D (1990). Competitive anxiety in sport.

[b25] Mellalieu SD (2003). Mood matters: but how much? A comment on Lane and Terry (2000). J Appl Sport Psychol.

[b26] Mellalieu SD, Hanton S, Shearer DA (2008). Hearts in the fire, heads in the fridge: a qualitative investigation into the temporal patterning of the precompetitive psychological response in elite performers. J Sports Sci.

[b27] Nicholls AR, Backhouse SH, Polman RCJ, McKenna J (2009). Stressors and affective states among professional rugby players. Scand J Med Sci Sports.

[b28] Owen T, Maynaryd I, Hanton S (2007). Intervening with athletes during the time leading up to competition: theory to practice II. J Appl Sport Psychol.

[b29] Prapavessis H, Grove RJ (1994). Personality variables as antecedents of precompetitive mood state temporal patterning. Int J Sport Psychol.

[b30] Rainey DW, Cunningham H (1988). Competitive trait anxiety in male and female college athletes. Res Q Exerc Sport.

[b31] Robazza C, Bortoli L, Nougier V (2000). Performance emotions in an elite archer: a case study. J Sport Behav.

[b32] Scollon CN, Kim-Prieto C, Diener E (2003). Experience sampling: promises and pitfalls, strengths and weaknesses. J Happiness Stud.

[b33] Smith CA, Lazarus RS (1993). Appraisal components, core relational themes, and the emotions. Cogn Emot.

[b34] Snijders TAB, Bosker RJ (1999). Multilevel analysis.

[b35] Thompson T, Altmann R, Davidson J (2004). Shame-proneness and achievement behaviour. Pers Indiv Diff.

[b36] Uphill MA, Jones MV (2007). Antecedents of emotions in elite athletes: a cognitive motivational relational theory perspective. Res Q Exerc Sport.

[b37] Vallerand RJ, Blanchard CM, Hanin YL (2000). The study of emotions in sport and exercise: historical, definitional, and conceptual perspectives. Emotions in sport.

[b38] Watson D (1988). Intraindividual and interindividual analyses of positive and negative affect: their relation to health complaints, perceived stress, and daily activities. J Pers Soc Psychol.

[b39] Weiner B (1985). An attributional theory of achievement motivation and emotion. Psychol Rev.

[b40] Wilson GV, Kerr JH (1999). Affective responses to success and failure: a study of winning and losing in competitive rugby. Pers Indiv Diff.

[b41] Youngstrom EA, Green KW (2003). Reliability generalization of self-report of emotions using the differential emotions scale. Educ Psychol Meas.

